# Açai (*Euterpe oleracea* Mart.) Upregulates Paraoxonase 1 Gene Expression and Activity with Concomitant Reduction of Hepatic Steatosis in High-Fat Diet-Fed Rats

**DOI:** 10.1155/2016/8379105

**Published:** 2016-08-25

**Authors:** Renata Rebeca Pereira, Isabel Cristina Mallosto Emerich de Abreu, Joyce Ferreira da Costa Guerra, Nara Nunes Lage, Juliana Márcia Macedo Lopes, Maísa Silva, Wanderson Geraldo de Lima, Marcelo Eustáquio Silva, Maria Lucia Pedrosa

**Affiliations:** ^1^Research Center in Biological Sciences, Federal University of Ouro Preto, 35400-000 Ouro Preto, MG, Brazil; ^2^Department of Biological Sciences, Federal University of Ouro Preto, 35400-000 Ouro Preto, MG, Brazil; ^3^Federal University of São Paulo, 04039-002 São Paulo, SP, Brazil; ^4^Postgraduate Program in Health and Nutrition, Federal University of Ouro Preto, 35400-000 Ouro Preto, MG, Brazil; ^5^Department of Basic Health, Federal University of Juiz de Fora, Governador Valadares Campus, 35010-177 Governador Valadares, MG, Brazil; ^6^Department of Foods, Federal University of Ouro Preto, 35400-000 Ouro Preto, MG, Brazil

## Abstract

Açai (*Euterpe oleracea *Mart.), a fruit from the Amazon region, has emerged as a promising source of polyphenols. Açai consumption has been increasing owing to ascribed health benefits and antioxidant properties; however, its effects on hepatic injury are limited. In this study, we evaluated the antioxidant effect of filtered açai pulp on the expression of paraoxonase (PON) isoforms and PON1 activity in rats with nonalcoholic fatty liver disease (NAFLD). The rats were fed a standard AIN-93M (control) diet or a high-fat (HF) diet containing 25% soy oil and 1% cholesterol with or without açai pulp (2 g/day) for 6 weeks. Our results show that açai pulp prevented low-density lipoprotein (LDL) oxidation, increased serum and hepatic PON1 activity, and upregulated the expression of PON1 and ApoA-I in the liver. In HF diet-fed rats, treatment with açai pulp attenuated liver damage, reducing fat infiltration and triglyceride (TG) content. In rats receiving açai, increased serum PON1 activity was correlated with a reduction in hepatic steatosis and hepatic injury. These findings suggest the use of açai as a potential therapy for liver injuries, supporting the idea that dietary antioxidants are a promising approach to enhance the defensive systems against oxidative stress.

## 1. Introduction

Nonalcoholic fatty liver disease (NAFLD) affects 10–30% of the general population, representing a spectrum of diseases ranging from simple steatosis to nonalcoholic steatohepatitis (NASH), NAFLD-associated cirrhosis, and end-stage liver disease [1]. NAFLD also increases the risk of hepatocellular carcinoma (HCC). HCC and end-stage liver disease may markedly increase the risk of liver-related mortality [2]. Oxidative stress is thought to be one of the underlying causes of NAFLD [3, 4]. Accumulation of lipids in hepatocytes impairs the oxidative capacity of the mitochondria, increasing the reduced state of the electron transport chain complexes and stimulating the peroxisomal and microsomal pathways of fat oxidation. As a consequence, reactive oxygen species (ROS) are generated that can induce lipid peroxidation, which in turn may be followed by inflammation and fibrogenesis [5].

Paraoxonase (PON) is associated with a variety of diseases involving oxidative stress [6]. The PON gene family contains three members, PON1, PON2, and PON3 [7, 8]. PON1 is a calcium-dependent esterase closely associated with apolipoprotein A-I (ApoA-I) and high-density lipoprotein (HDL) and has been reported to confer antioxidant properties by decreasing the accumulation of lipid peroxidation products [9, 10]. PON1 prevents the production and accumulation of lipoperoxides in low-density lipoprotein particles (LDL) [11, 12] and protects phospholipids from oxidation in HDL. The liver plays a key role in the synthesis of serum PON1, which hydrolyzes a number of substrates, including paraoxon, phenyl acetate, lipid peroxides, and hydroperoxides [13]. PON3 is also predominantly expressed in the liver and secreted into the plasma, where it has an antioxidant effect against LDL oxidation [14]. PON2 is not detectable in serum but is expressed in many tissues, including brain, liver, kidney, and testis [15], and plays a role in decreasing cellular oxidative stress and the inflammatory response.

PON1 activity is reduced in many diseases such as atherosclerosis [16], diabetes mellitus [17], chronic kidney disease [18], and hypercholesterolemia [19]. Deficient serum PON1 activity has been associated with an increased risk for Alzheimer's disease [20]. In addition, an active role of PON1 in the regulation of oxidative stress, fibrosis, and hepatic cell apoptosis in chronic liver diseases has been suggested [21–23]. PON1 levels significantly decreased in serum of patients with chronic liver diseases such as NAFLD, hepatitis, and cirrhosis [13, 24]. More relevantly, PON1-deficient mice fed a high-fat high-cholesterol diet showed histological alterations in the liver, suggesting that PON1 plays a major role in protection against oxidative stress on diet-induced fatty liver [25].

Dietary polyphenols, including resveratrol and flavonoids, such as quercetin and curcumin, upregulate PON1 [26, 27]. Recent studies have indicated the sterol regulatory element-binding proteins (SREBPs) as targets of polyphenols [28, 29]. SREBPs play a central role in cellular lipogenesis and lipid homeostasis by controlling the synthesis of fatty acids, triacylglycerols, and cholesterol [30] and comprise three main proteins, termed SREBP-1a, SREBP-1c, and SREBP-2, that are encoded by two genes: SREBP1 and SREBP2. While SREBP-1a and SREBP-1c control the fatty acid pathways, SREBP-2 regulates the cholesterol biosynthetic pathway. It was demonstrated that quercetin upregulates PON1 gene transcription and PON1 activity via SREBP-2 that translocates from the endoplasmic reticulum to the nucleus, where it specifically interacts with sterol responsive-like sequence in PON1 promoter [31]. Polyphenols also might activate the aryl hydrocarbon receptor (AhR), a transcription factor sensor for organic chemicals, and stimulate PON1 transcription activation [32, 33].

Açai (*Euterpe oleracea* Mart.), a fruit native to the Amazon region, has gained international attention as a functional food owing to its high content of polyphenols and potential health benefits. Most of the beneficial effects of açai are attributed to secondary metabolites such as flavonoids and other polyphenols, including anthocyanins and proanthocyanidins (specifically cyanidin-3-glucoside and cyanidin-3-rutinoside), which provide antioxidant activity [34, 35]. Previous* in vitro* and animal model studies showed that açai consumption slows the progression of oxidative stress [36, 37] and induces hypocholesterolemic [38], antiatherogenic, anti-inflammatory [39], and hepatoprotective [40] effects. Previous work from our laboratory showed that diet supplementation with açai increased serum activity of the antioxidant enzyme PON1 in rats [19] but did not evaluate the effect of açai on the expression of PON isoforms.

In spite of these advances, we still do not know whether açai treatment can affect liver PON isoforms and influence the progression of NAFLD in rats. Therefore, in this study we evaluated the protective effects of açai against oxidative stress induced by HF diet with respect to LDL oxidation, expression of PON isoforms, and PON1 activity in rats with NAFLD. Our results show that açai protected LDL against oxidation and at the same time increased serum and hepatic PON1 activity and upregulated the expression of PON1 and ApoA-I in the liver. Adding to these effects, açai concomitantly ameliorated hepatic steatosis and hepatic injury. Because exogenous antioxidant sources have been shown to help retard NAFLD progression [41], we believe that the results presented herein may contribute to future efforts researching açai and other polyphenol-rich foods as a potential therapy for liver injuries and other degenerative diseases.

## 2. Materials and Methods

### 2.1. Chemicals and Reagents

2,2-Diphenyl-1-picrylhydrazyl (DPPH), 6-hydroxy-2,5,7,8-tetramethylchromane-2-carboxylic acid (Trolox), gallic acid, thiobarbituric acid (TBA), trichloroacetic acid (TCA), 1,1,3,3-tetramethoxypropane, phenyl acetate, butylhydroxytoluene (BHT), Tris(hydroxymethyl)aminomethane, dithiothreitol (DTT), and protease inhibitor cocktail were purchased from Sigma-Aldrich (St. Louis, MO, USA). Triton-X100 and Folin-Ciocalteu phenol reagent were purchased from VETEC (Duque de Caxias, Rio de Janeiro, Brazil). Chloroform, methanol (MeOH), calcium chloride (CaCl_2_), and glycerol were purchased from Synth (Diadema, São Paulo, Brazil). RNAgents Total RNA Isolation System was purchased from Promega Corporation (Madison, WI, USA). High-Capacity cDNA Reverse Transcription Kit and Power SYBR® Green PCR Master Mix reagent were purchased from Applied Biosystems (Foster City, CA, USA), Rat Ox-LDL ELISA kit (Cat. number E-EL-R0710) was purchased from Elabscience Biotechnology Co., Ltd. (Wuhan, China), and kits for biochemical analysis were purchased from Labtest Diagnostica SA (Lagoa Santa, MG, Brazil).

### 2.2. Açai Pulp Preparation and Composition

A single lot of pasteurized frozen açai pulp without colorants or preservatives was obtained from Icefruit Comércio de Alimentos Ltda. (Tatuí, São Paulo, Brazil). The pulp was stored at −20°C until use, when it was thawed and sieved through a 22-mesh sieve. The resultant filtered açai pulp was directly administered to animals by oral gavage. The macronutrient composition of filtered açai pulp was as follows (per 100 g): 96 g moisture, 1.196 g lipids, 0.059 g carbohydrates, and 0.416 g proteins, all determined according to the Association of Official Analytical Chemists [42], and 2.202 g neutral detergent fiber, determined according to Van Soest and Wine [43]. The total caloric content of the filtered açai pulp was 12.7 kcal/100 g.

### 2.3. Phytochemical Composition and DPPH Radical-Scavenging Assay

Total phenolic content of filtered açai pulp was determined by colorimetric analysis using the Folin-Ciocalteu reagent, as described by Georgé et al. [44]. Briefly, 0.5 mL of the diluted sample or of a standard solution of gallic acid was added to 2.5 mL of 1 : 10 diluted Folin-Ciocalteu reagent. After 2 min at room temperature, 2 mL of saturated sodium carbonate solution (7.5%) was added and mixed vigorously. After incubation at 50°C for 15 min, the mixture was placed in an ice bath. Absorbance at 760 nm relative to the blank was determined. The obtained measurement was compared to a gallic acid calibration curve, and results were expressed in milligrams of gallic acid equivalents (GAE) per 100 g of filtered pulp.

Total monomeric anthocyanin content of filtered açai pulp was determined by the differential pH method as described previously [45] and modified by Guerra et al. [36]. Samples were diluted with two different buffers: potassium chloride (0.025 M), pH 1.0, and sodium acetate (4.0 M), pH 4.5. Absorbance was determined simultaneously as absorption maxima for the visible light spectrum and at 700 nm after incubation in the dark for 30 min at room temperature. Total anthocyanin content was expressed in milligrams of cyanidin-3-glucoside equivalents per 100 g of filtered pulp. A molar absorptivity of 26 900 M^−1^ cm^−1^ and a molecular mass of 449.2 g/mol were used for cyanidin-3-glucoside.

Measurement of DPPH radical-scavenging activity was determined by the method of Brand-Williams et al. [46] with modifications. Briefly, a 100 *μ*L aliquot of filtered açai pulp was added to 3.9 mL of a MeOH solution containing 60 *μ*M DPPH. The mixture was homogenized and left to stand at room temperature for 30 min in the dark. Absorbance was measured spectrophotometrically at 515 nm against a MeOH blank. Antioxidant activity was determined by the reduction in the absorbance of the DPPH radical. Trolox was used as the antioxidant standard. The percentage of inhibition was determined according to the formula = (1 − *A*_Sample  515_/*A*_Control  515_) × 100, where *A*_Sample  515_ and *A*_Control  515_ represent the absorbance of sample and control, respectively, at 515 nm. The results are expressed as mean ± SEM (standard error of the mean) from 3 replicated measurements.

### 2.4. Animals

Rats* Fischer* 344 (F344) were used. The animals were kindly supplied by Dr. Enio Cardillo Vieira, Gnotobiology Laboratory, Federal University of Minas Gerais, ICB, Brazil. Some of these animals were maintained in our laboratory (Bioterium of Experimental Nutrition Laboratory, School of Nutrition, Federal University of Ouro Preto, MG, Brazil) for many generations. Nine-week-old female rats weighing 140 ± 2 g were used in this study. The animals were individually housed in wire-bottomed cages and maintained in a room with controlled conditions (22 ± 2°C, 55% humidity, and 12 h light/dark cycles), and food and filtered water were provided* ad libitum* for 8 weeks. All animal procedures were approved by the Ethics Committee in Animal Research of the UFOP (Protocol number 2012/17).

### 2.5. Experimental Design and Diets

The animals were weighed one by one and placed in individual cages. After weighing 32 rats, mean weight was calculated, and if the difference between the average weights was ≤2 g, the experiment was started. The rats (*n* = 32) were divided into 2 experimental groups of sixteen animals each. The first group served as the control (C) and received a standard AIN-93M diet [47]. The second group (HF) received a high-fat diet (containing 25% soy oil and 1% cholesterol) [19, 48]. After two weeks, the C group rats were subdivided into C and CA experimental groups with eight animals each, balanced for weight; and the HF group were subdivided into HF and HFA experimental groups with eight animals each, balanced for weight. The C and CA groups received the same standard diet, and H and HFA groups received the same HF diet. The CA and HFA groups were treated with filtered açai pulp, administered as a single dose (2 g/day) via gavage during the light phase for 6 weeks. The dose of açai used in this study mimics the consumption of a portion of this fruit in the human diet and corresponds to adding 2% of açai pulp to rat diet considering the rat daily average intake, as per previous studies [19, 38]. This dose had already been shown to have beneficial effects in experimental models [19, 36, 38, 49]. The C and HF groups received equal volumes of distilled water. The experimental diets were prepared in the Laboratory of Experimental Nutrition, School of Nutrition, Federal University of Ouro Preto, MG, Brazil, from semipurified ingredients and stored at 4°C. The compositions of the diets are described in Table 1.

### 2.6. Sample Preparation

At the end of the experimental period, the rats were fasted for 12 hours, anesthetized with isoflurane, and euthanized by total blood collection from adjacent vessels to the brachial plexus. Blood samples were collected in polypropylene tubes and centrifuged at 3000 ×g for 15 min.

Afterwards, the serum was removed and stored at −80°C. The liver was collected, washed in saline, and weighed. The small hepatic lobe was stored in buffered formalin for histopathological analysis and the rest of the liver was submerged in liquid nitrogen and immediately stored at −80°C for subsequent analysis.

### 2.7. Serum Biochemical Analysis

Serum activities of alanine aminotransferase (ALT) and aspartate aminotransferase (AST) were measured enzymatically using kits from Labtest.

### 2.8. Measurement of Ox-LDL

Ox-LDL levels were measured in a 96-well plate by a sandwich ELISA method using a kit from Elabscience. For the assay, 100 *μ*L of standard and diluted serum was added to wells that were coated with antibodies before incubation at 37°C for 90 min. The residual antigens that did not bind with antibodies were washed away. After that, biotin-antibodies were added into each well and incubated at 37°C for 1 h. The fluid in the wells was aspirated and the wells were washed three times. Next, 100 *μ*L HRP-avidin was added into each well and incubated at 37°C for 30 min. The aspiration step was repeated and the wells were washed again five times. TMB substrate (90 *μ*L) was added to each well and then incubated at 37°C for 15 min protected from light. To stop the reaction, 50 *μ*L of stop solution was added and absorbance was read immediately at 450 nm.

### 2.9. Determination of Liver Lipids Levels

Hepatic lipids were extracted from liver tissue using a chloroform/MeOH solution (2 : 1, v/v), as described by Folch et al. [50]. The content of total lipids in the liver was quantified gravimetrically by evaporation of the solvents and dissolution of the dried lipids in 1 mL isopropanol. Triacylglycerols (TG) in this solution were determined using a kit from Labtest.

### 2.10. Liver Lipid Peroxidation

Lipid peroxidation was assessed by the thiobarbituric acid-reactive substances (TBARS) assay described by Buege and Aust [51]. Liver tissues (100 mg) were homogenized in Tris/HCl buffer (20 mM) and kept on ice. Homogenate (500 *μ*L) was transferred to tubes, mixed with 250 *μ*L of TCA (28% w/v in HCl 0.25 N), 250 *μ*L of TBA (1% acetic acid 0.25 N), and 125 *μ*L of BHT (125 mM in ethanol), and vortexed. The tubes were heated at 95°C for 15 min and placed in an ice bath and, after the addition of 0.6 mL butanol, centrifuged at 10 000 ×g for 10 min at 4°C. The top phase of each solution was transferred to a 96-well plate and the absorbance was measured at 535 nm. The concentration of TBARS was calculated using 1,1,3,3-tetramethoxypropane as a standard. The concentration of total protein in the sample was determined by the Lowry assay [52]. Results are expressed as nmol of malondialdehyde (MDA)/mg protein.

### 2.11. Histological Examination

Liver tissue fragments were fixed in 10% buffered formalin for 72 h, after which the fragments were dehydrated, cleared, and embedded in paraffin. Tissue sections (4 *μ*m) were cut with a microtome (Leica, Germany) and mounted on microscope slides. The slides were then stained with hematoxylin and eosin (H&E) and photographed at 400x magnification (Leica Application Suite, Germany). Liver histology was examined using 15 images obtained at random from the tissue and classified for the degree of macrovesicular steatosis. Ten low-power fields were examined in each of the images and the degree of lipid infiltration was graded with a semiquantitative score reflecting the percentage of hepatocytes containing lipid droplets. Steatosis scores of <33% of cells, 33–66% of cells, and >66% of cells were classified as grade I, grade II, and grade III, respectively [53].

### 2.12. Preparation of Microsomal Fractions

Microsomal fractions were prepared by a modification of the method of Bayrak et al. [54]. Frozen livers were homogenized in (1 : 4, w/v) ice-cold 5 mM Tris/HCl (pH 7.4) buffer containing 0.9 mM CaCl_2_, 1 mM DTT, 10% glycerol, 10 mM NaCl, and 5 *μ*L/mL protease inhibitor cocktail. Fractionation of the sample was performed according to Cox and Emili [55] with modifications. The homogenate was centrifuged at 800 ×g for 15 min at 4°C for nuclei removal, followed by centrifugation of supernatant at 6000 ×g for 20 min at 4°C for separation of the mitochondrial pellet. The pellet was discarded and the ultracentrifugation of the supernatant was performed at 80 000 ×g for 1 h at 4°C. The resulting microsomal pellet was resuspended in 9 mM Tris/HCl buffer (pH 8.0) containing 2 mM CaCl_2_. Solubilization of microsomal membranes was achieved by the addition of Triton X-100 at a final concentration of 0.1% and gentle stirring of the sample for 40 min. Subsequently, the sample was kept on ice for 30 min and recentrifuged at 80 000 ×g for 1 h at 4°C, after which the supernatants were collected. The concentration of total protein in the fractions was determined by the Lowry assay [52].

### 2.13. Serum and Liver PON1 Activity Measurement

Serum and liver PON1 enzyme activity was measured as described by Bełtowski et al. [56], with phenyl acetate as the substrate in an aliquot of serum or the microsomal suspension from the liver based on the initial rate of substrate hydrolysis to phenol, whose absorbance was monitored at 270 nm. The assay mixture contained 5 *μ*L of serum (diluted 1 : 3) or microsomal suspension in 2.5 mL of 9 mM Tris/HCl buffer (pH 8.0) containing 2 mM CaCl_2_ and 1 mM phenyl acetate. The results were calculated assuming the molar extinction coefficient of phenyl acetate to be 1310 L mol^−1^ cm^−1^. The values were expressed in units per milliliter of serum or per gram equivalent of liver microsomes, where 1 U of arylesterase hydrolyzes 1 mmol of phenyl acetate per minute.

### 2.14. Real-Time Quantitative Reverse Transcription Polymerase Chain Reaction (RT-PCR) Assay

Total RNA was extracted from liver samples using the RNAgents Total RNA Isolation System according to the manufacturer's instructions. cDNA was synthesized from 2 *μ*g of total RNA with random primers using the High-Capacity cDNA Reverse Transcription Kit and following the manufacturer's recommendations. Real-time PCR was performed using the Power SYBR Green PCR Master Mix reagent.

The sequences of oligonucleotide primers for PCR included 18S rRNA: 5′-GTAAGTGCGGGTCATAAG-3′ (forward); 5′-CCATCCAATCGGTAGTAGC-3′ (reverse); PON1: 5′-AAGCTGGCTACACCCACATC-3′ (forward); 5′-CAACATTCGTTGGTGAGCGG-3′ (reverse); PON2: 5′-TTCTTCAGGCGACATCTGGG-3′ (forward); 5′-TCTGACGAGGGATGGTT-3′ (reverse); PON3: 5′-AAGCTTTGCACCAGACAAGC-3′ (forward); 5′-GTCCTGGTCGAACCCATCAC-3′ (reverse); APOA-1: 5′-TTGGTCGCCTACAGGAACAG-3′ (forward); 5′-TGGAATTCATCCAGGTGGGG-3′ (reverse); AHR: 5′-GCCAATACGCACCAAAAGCA-3′ (forward); 5′-TCGTCCTGTTGGATCAAGGC-3′ (reverse); SREBP-2: 5′-AGCTGGCAAATCAGAAAAACAAG-3′ (forward); 5′-GATTAAAGTCTTCAATCTTCAAGTCCAC-3′ (reverse). The PCR conditions were as follows: 50°C for 2 min, 95°C for 10 min, 40 cycles of 95°C for 15 s (denaturation), and 60°C for 1 min (primer annealing and product extension). The specificity of the products obtained was confirmed by analysis of the amplified product dissociation curves. The data obtained were analyzed using the comparative Cq method. Target gene expression was determined relative to the expression of the endogenous 18S ribosomal RNA gene. All analyses were performed in triplicate.

### 2.15. Statistical Analysis

Statistical analysis was performed using GraphPad Prism 5.00 version for Windows (GraphPad Software, San Diego, CA). The normality of the data was tested using the Kolmogorov-Smirnov test. The data were analyzed by one-way analysis of variance (ANOVA), followed by Tukey's* post hoc* test or the Kruskal-Wallis test and the Dunn posttest (for parametric and nonparametric data, resp.). Results were considered statistically significant for *p* values < 0.05. All data were expressed as the mean ± standard error of the mean (SEM) or as medians and interquartile ranges. Correlations were calculated using Pearson's and Spearman's correlation coefficients to measure the degree to which two variables were related.

## 3. Results

### 3.1. Polyphenols and* In Vitro* Antioxidant Capacity of Açai

The total polyphenol and total monomeric anthocyanin content of filtered açai pulp were 458.6 mg GAE/100 g and 13.59 mg/100 g, respectively. The ability of three different concentrations of filtered açai pulp (20, 40, and 100 mg/mL) to neutralize the DPPH radical was determined. As presented in Table 2, the filtered pulp displays increasing* in vitro* antioxidant capacity in a dose-dependent manner. All the concentrations tested showed a high neutralization capacity, similar to the standard antioxidant Trolox in the range of 100 to 700 *μ*M/L.

### 3.2. Effect of Açai on Liver Injury and Hepatic Steatosis

**Figure 1:**
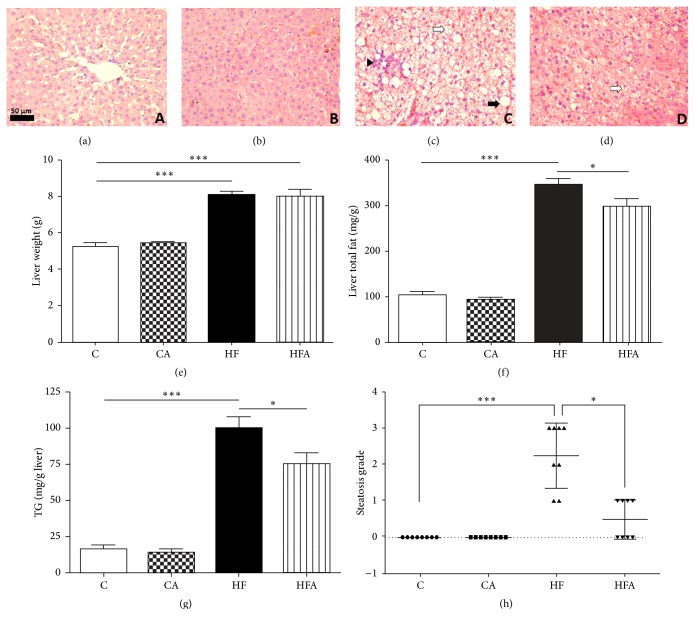
Hematoxylin and eosin staining of representative sections of livers from rats fed a control or high-fat diet treated with açai pulp. Hepatocytes abnormalities were not observed in the C group (a) and CA group (b). Severe hepatic steatosis was observed in the HF group (c) with the presence of macro- and microvesicular steatosis and inflammatory infiltrate. Steatosis and fat accumulation were reduced in the HFA group (d). Macrovesicular injury is indicated by the black arrow, microvesicular injury by white arrows, and inflammatory infiltrate by the black arrowhead. Images were photographed at 400x magnification. Scale bar = 50 *μ*m. Liver weight (e), liver total fat (f), hepatic triacylglycerol content (g), and grade of liver steatosis (h) of rats fed with control or high-fat diet treated with açai. Data in (e), (f), and (g) were represented as mean ± SEM (*n* = 8). ^*∗*^ *p* < 0.05 and ^*∗∗∗*^ *p* < 0.001 by ANOVA test followed Tukey's posttest. Data in (h) were represented as median and interquartile range (*n* = 8). ^*∗*^ *p* < 0.05 and ^*∗∗∗*^ *p* < 0.001 by Kruskal-Wallis test followed by Dunn's posttest.

At the end of the experimental period, rats fed an HF diet exhibited an increase in the activity of serum enzymes ALT and AST (*p* < 0.001 and *p* < 0.05, resp.) (Table 3). Furthermore, liver histology showed increased lipid deposition in the livers of the HF rats (Figure 1(c)), characterized by accentuated micro- and macrovesicular fat droplets. In the same group, infiltration of inflammatory cells into the liver can be seen. Hepatocytes abnormalities were not observed in the C group and CA group (Figures 1(a) and 1(b), resp.). Animals belonging to the HF group also had a higher steatosis score (*p* < 0.001) compared to those belonging to the C group (Figure 1(h)). Açai pulp administration promoted a 30% reduction in the serum ALT activity in HFA rats compared to that in HF group (*p* < 0.05) (Table 3). Indeed, açai pulp treatment relieved hepatic steatosis, as evidenced by the occurrence of mild steatosis, lower steatosis score, and less lipid-loaded hepatocytes in HFA rats (Figures 1(d) and 1(h)) compared to that in HF group (Figures 1(c) and 1(h)).

The HF and HFA groups also showed significantly higher liver weight, which was consistent with the significant increase of liver total fat and TG content in the same groups as compared to control groups (*p* < 0.001) (Figures 1(e), 1(f), and 1(g)). Livers from animals fed an HF diet and treated with açai pulp had 13.89% lower total fat and 24.45% lower TG content compared to those of animals fed the HF diet alone (Figures 1(f) and 1(g)).

### 3.3. Effect of Açai on Serum LDL Oxidation and Liver Lipid Peroxidation

**Figure 2:**
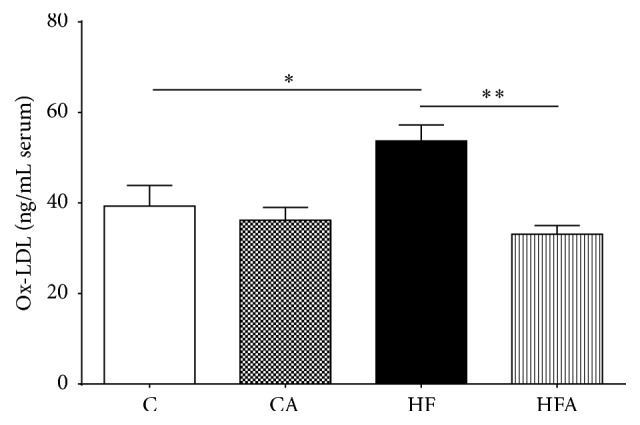
Oxidized LDL levels in serum of rats fed a control or high-fat diet treated with açai pulp. Values were expressed as means ± SEM (*n* = 6). ^*∗*^ *p* < 0.05 and ^*∗∗*^ *p* < 0.01 by ANOVA test followed Tukey's posttest.

As shown in Figure 2, HF group showed significantly increased serum ox-LDL concentration compared to C group (*p* < 0.05). Administration of açai pulp for 6 weeks promoted a 39% reduction in the ox-LDL levels in the HFA group in comparison with the HF group (*p* < 0.01).

Additionally, to estimate the extent of lipid peroxidation in the liver, TBARS were determined. Compared to control animals, HF rats showed a 1.4-fold increase in TBARS levels (*p* < 0.05). No statistical difference was found between the HF and HFA animals; however, the HFA group was not different to the control group (Table 3).

### 3.4. Effect of Açai on Expression of PON Isoforms mRNA and Genes Related to PON1 Regulation in the Liver

**Figure 3:**
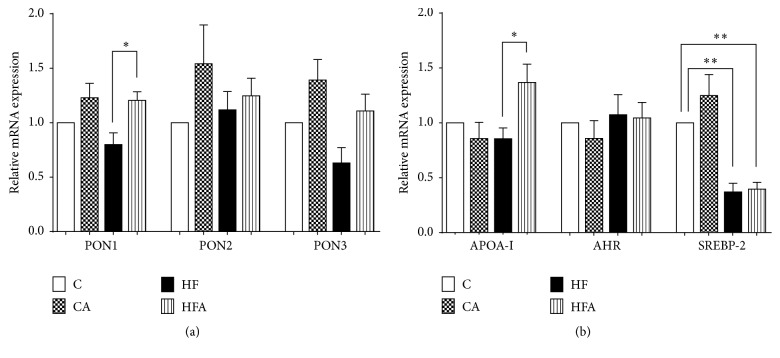
Relative expression of mRNA of genes of three isoforms of the enzyme paraoxonase PON1, PON2, and PON3 (a) and of genes APOA-1, AHR, and SREBP-2 (b) in rats fed a control or high-fat diet treated with açai pulp. Values are relative to the expression level of the 18S rRNA gene and were normalized by the control group. Values were expressed as means ± SEM (*n* = 6). ^*∗*^ *p* < 0.05 and ^*∗∗*^ *p* < 0.01 by ANOVA test followed Tukey's posttest.

As shown in Figure 3(a), the HFA group exhibited approximately 1.5-fold increase in the expression of PON1 mRNA in comparison with the HF group (*p* < 0.01). Moreover, in HFA animals, there was a trend toward increased liver PON3 mRNA expression compared to the HF group, although it was not significant. There was no difference in the expression of PON2 mRNA among the experimental groups.

Concerning the expression of APOA-1, AHR, and SREBP-2 mRNA (PON1 regulation-related genes), a significant increase in the hepatic mRNA expression of APOA-1 in HFA rats occurred, compared to that in HF rats (*p* < 0.05) (Figure 3(b)). Animals fed the HF diet presented a decrease in SREBP-2 mRNA expression compared to control animals (*p* < 0.01). The expression of SREBP-2 was not affected by açai treatment. No significant changes in the expression of AHR mRNA were found among the experimental groups (Figure 3(b)).

### 3.5. Effect of Açai on Serum and Hepatic PON1 Activity

**Figure 4:**
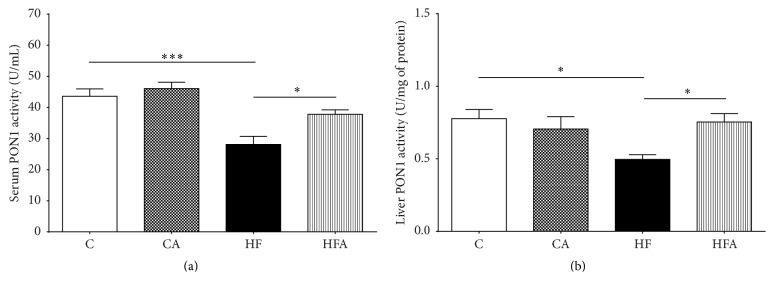
PON1 arylesterase activity in serum (a) and liver (b) of rats fed a control or high-fat diet treated with açai pulp. Values were expressed as means ± SEM (*n* = 8). ^*∗*^ *p* < 0.05 and ^*∗∗∗*^ *p* < 0.001 by ANOVA test followed Tukey's posttest.

As shown in Figures 4(a) and 4(b), both serum and hepatic arylesterase activity of PON1 were significantly lower in the HF group than in both control groups (*p* < 0.001 and *p* < 0.05, resp.). Administration of açai pulp for 6 weeks promoted a 34% increase in the arylesterase activity of PON1 in serum and a 52% increase in the arylesterase of PON1 activity in the liver of HFA rats compared to that in HF rats (*p* < 0.05).

### 3.6. Relationship between Serum and Hepatic PON1 Activity and Liver Disease Variables

**Figure 5:**
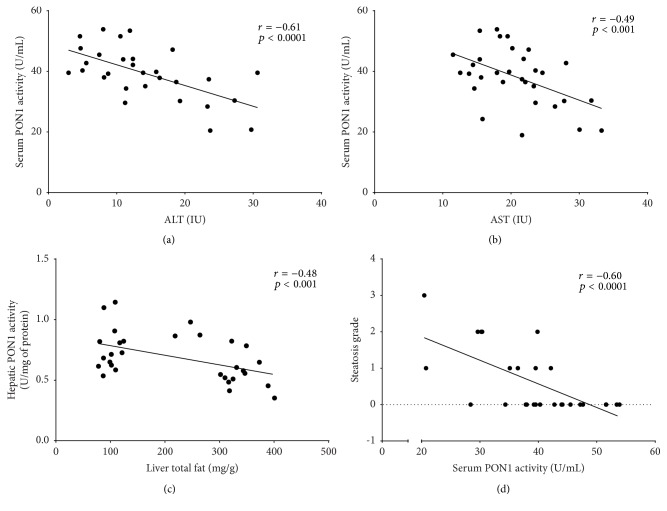
Correlation between serum and hepatic PON activities and variables in the rat nonalcoholic fatty liver disease experimental model: (a) correlation between serum PON1 and ALT activities; (b) correlation between serum PON1 and AST activities; (c) correlation between hepatic PON1 activity and total liver fat; (d) correlation between serum PON1 activity and the grade of steatosis.

Pooling the data from all groups, we found a negative correlation between serum PON1 and ALT and AST activities (*r* = −0.61, *p* < 0.0001 and *r* = −0.49, *p* < 0.001, resp.) (Figures 5(a) and 5(b)). Moreover, hepatic PON1 activity was negatively correlated with the amount of total fat extracted from the liver (*r* = −0.48, *p* < 0.001) (Figure 5(c)) and serum PON1 activity was negatively correlated with the degree of steatosis (*r* = −0.60, *p* < 0.0001) (Figure 5(d)).

## 4. Discussion

In this study, we examined the effects of filtered açai pulp on the progression of high-fat diet-induced fatty liver in rats. We provide evidence that the treatment with açai pulp, a source of polyphenols, prevents LDL oxidation by increasing serum and hepatic PON1 activity and mRNA expression. The treatment with açai also alleviated the severity of hepatic steatosis in rats fed a high-fat diet. Moreover, in the same animals, we observed a treatment effect on the increased ApoA-I mRNA expression levels.

Açai contains various bioactive secondary metabolites, mostly phenolic acids and flavonoids, which have been identified as potential antioxidants [57, 58]. As expected, our filtered açai pulp contained high levels of total phenolic compounds and total monomeric anthocyanins, which probably explain its high antioxidant capacity, as suggested elsewhere [59–61]. In addition to polyphenols, other nutrient fractions in açai pulp may exert beneficial biological effects. Among the constituents of açai pulp are very high amounts of fiber [34]. Although the amount of fiber was reduced in our filtered açai pulp, it was comparable to the açai pulp ingested by humans in another study [62]. Many of the health benefits ascribed to fiber are a consequence of fermentation by colonic microbiota and the secondary metabolites that are produced. There is mounting evidence that these metabolites may play a key role in the prevention and management of diseases [63].

Several studies have connected decreased PON1 activity with hepatic injury, including NAFLD [24, 64, 65]. PON1 is known to attenuate oxidative stress, which is augmented during steatosis progression [13, 66]. Our results show that rats fed a high-fat diet developed hepatic steatosis, evidenced by intense TG accumulation and micro- and macrovesicular fat droplets. In addition, the HF rats had increased oxidation of LDL in serum and TBARS levels in liver. The HF rats also showed reduced serum and liver PON1 activity. These findings corroborate with García-Heredia et al. [25] that showed that PON-deficient mice fed a high-fat high-cholesterol diet presented significant alterations in liver tissues, such as increased hepatic steatosis and the expression of makers of oxidative stress and inflammation.

There is a growing body of evidence that oxidized LDL (ox-LDL) may play a role in the pathophysiology of NAFLD. LDL oxidation is a well-known event in the development of atherosclerosis [67] and NAFLD has many features in common with cardiovascular diseases, including lipid accumulation, macrophage activation and infiltration, and inflammation. Recent animal studies have suggested that dietary cholesterol is a critical factor in induction of NAFLD and showed that Ldlr^−/−^ mice are more vulnerable to cholesterol-induced NAFLD than wild-type mice [68, 69]. One proposed theory points to an intrinsic defective mechanism of lipid traffic within the Kupffer cells, the resident liver macrophages. The activation of Kupffer cells by ox-LDL leads to a rapid release of various inflammatory mediators and signaling molecules such as cytokines, ROS, proteases, and lipid mediators and contributes to hepatic inflammation [70]. Similar to the formation of foam cells in atherosclerosis, hyperlipidemic mice showed hypertrophy of Kupffer cells, which was correlated with liver inflammation in NAFLD [71]. In the present study, we demonstrated that filtered açai pulp protected LDL against oxidation and at the same time upregulated PON1 activity. Based on these findings, we propose that açai has a role in the regulation of PON1 activity and expression, which reduces the production and accumulation of lipoperoxides in LDL, leading to improvement of hepatic steatosis.

Dietary polyphenols such as those found in açai may play an important role in improving the antioxidant status, since they neutralize oxygen-reactive species (ROS), act as chelators of metal ions, and modulate the activity of enzymes, including PON [72–74]. A recent study involving women showed that the daily intake of 200 g of açai pulp for four weeks improved the antioxidant status by increasing the activity of the enzyme catalase and total antioxidant capacity (TAC) in polymorphonuclear (PMN) cells and decreasing the production of ROS [62]. Resveratrol, a polyphenolic phytoalexin found in grapes and wine, was shown to increase PON1 activity levels in 24-month-old obese rats [75] and to increase PON1 gene expression in human hepatocyte primary cultures and in HuH7 cells [32]. Although isolated compounds have been explored as a potential nutritional therapy for a large number of diseases, our findings demonstrate that the açai pulp possesses important biological effects: improving the antioxidant status and alleviating NAFLD. Açai pulp may produce more pronounced effects than a single isolated compound, as crude extracts can exert additive and synergistic effects attributed to the complex mixture of phytochemicals [76].

Our group has previously shown that dietary açai pulp modulates gene expression of hepatic enzymes in diabetic rats and increases serum activity of PON1 in a hypercholesterolemic rat model [19, 36]. However, that study did not assess the expression of PON isoforms. In mice, Guerra et al. [40] demonstrated that administration of açai extract reduced hepatic steatosis by modulation of pathways related to hepatic lipid metabolism but did not evaluate possible effects on antioxidant enzymes.

In this study, we demonstrated that açai treatment increased serum and hepatic PON1 activity and PON1 mRNA expression. We did not find a significant treatment effect on the expression of other isoforms of PON. At the same time, we observed that açai treatment of rats fed with an HF diet protected against liver damage and reduced liver TG content, serum ALT enzyme activity, and the degree of steatosis, as confirmed by the decline in the number of macrovesicular vacuoles. We also found a negative correlation between serum PON1 activity and progression of steatosis in rats receiving açai pulp. Our results corroborate the report of Guerra et al. [40] and therefore strengthen the hypothesis that açai acts by retarding oxidative stress-associated liver damage induced by HF diet.

Factors known to influence PON1 activity and/or concentration include PON1 interaction with oxidized lipids and/or other oxidants [77, 78]. Other mechanisms by which polyphenols affect PON1 activity may be present. Studies have reported upregulation of hepatocyte PON1 mRNA expression and activity by polyphenols in an aryl hydrocarbon receptor- (AhR-) dependent manner via activation of a xenobiotic responsive element-like sequence [32, 79]. In addition, the sequence in the PON promoter region containing the binding site for the transcription factor sterol regulatory element-binding protein-2 (SREBP-2) has also been shown to be a target for upregulation of PON1 [80]. In our study, açai treatment did not change the gene expression levels of AHR. In addition, we demonstrated that rats fed with an HF diet exhibited a lower expression of SREBP-2 and that açai treatment did not restore these levels. These findings do not support the hypothesis that açai can activate the PON transcription via activation of these pathways. More investigation of the targets of these pathways may help elucidate the mechanisms by which açai regulates PON1 expression and activity.

As known, the PON1 enzyme serum levels are strongly associated with apolipoprotein A-I (ApoA-I) in HDL. ApoA-I is synthesized in the liver and to a lesser extent in the intestine [81]. The role of ApoA-I and PON1 in inhibition of atherosclerosis has been reported [82]. In this study, we found that açai pulp treatment upregulated the expression of ApoA-I mRNA. Moore et al. [83] showed that ApoA-I deficiency provoked a reduction in PON1 activity and did not alter the levels of HDL in C57BL/6 ApoA-I^−/−^ mice. Moreover, it was demonstrated that C57BL/6 ApoA-I^−/−^ mice developed NAFLD, suggesting that ApoA-I can be an important modulator of processes associated with deposition of lipids in the liver and development of diet-induced NAFLD. Our findings reinforce the notion of antiatherogenic effects of açai, as suggested elsewhere [84].

In summary, previous studies and the results presented here suggest that the hepatoprotective role of açai in the development of NAFLD is partly mediated by PON1, an antioxidant enzyme also involved in diseases such as hypercholesterolemia [19], atherogenesis [11], and neurological disorders [85]. In the context of the increased interest in and demand for açai in recent years, attributable to evidence that its use is associated with several beneficial health effects, the present study adds to our current understanding of the biological effects of açai on NAFLD.

## 5. Conclusion

Our data reveal that dietary açai improves response to oxidative stress by upregulating PON1 and prevents LDL oxidation, exerting a protective role in the progression of NAFLD. These findings indicate that açai is suitable as a potential nutritional therapy and can possibly be of benefit in prevention of diseases such as NAFLD and atherosclerosis. This study provides new perspectives for the understanding of the antioxidant effect of this fruit. Future epidemiological studies aimed at populations in regions that consume significant amounts of açai pulp in their diet should be undertaken to determine if these populations show a lower incidence of NAFLD, NASH, or end-stage liver disease.

## Figures and Tables

**Table 1:** Composition of the experimental diets (g/kg diet).

Nutrients	Diets	
	Standard diet (AIN-93M)	High-fat diet
Casein	140.0	140.0
Corn starch	622.5	402.5
Soybean oil	40.0	250.0
Cholesterol	—	10.0
Choline	2.5	2.5
Mineral mixture^a^	35.0	35.0
Vitamins mixture^b^	10.0	10.0
Cellulose	50.0	50.0
Saccharose	100.0	100.0
Energy content (kcal/kg)^c^	3810	4820

^a^Mineral and ^b^vitamins mixture as recommended by the AIN-93M rodent diet.

^c^Conversion factors: protein, 4 kcal/g; fat, 9 kcal/g; sugars, 4 kcal/g.

**Table 2:** Phytochemicals and antioxidant capacity of filtered açai pulp.

Compounds	Concentration	% of inhibition
Total phenolic (mg GAE/100 g)	458.60 ± 4.20	—
Total anthocyanins (mg/100 g)	13.59 ± 0.19	—

*DPPH radical-scavenging activity*		
*Filtered açai pulp*		
100 mg/mL	—	82.68 ± 0.23
40 mg/mL	—	54.67 ± 1.09
20 mg/mL	—	32.23 ± 0.34

*Trolox* ^a^		
700 *µ*M/L	—	50.9
300 *µ*M/L	—	21.23
100 *µ*M/L	—	9.64

^a^TEAC, Trolox equivalent antioxidant capacity. DPPH values of AAE are expressed as mean ± SEM and Trolox values are expressed as mean. Total phenolics are expressed in milligrams of gallic acid equivalents (GAE) per 100 g of filtered açai pulp.

**Table 3:** Effect of filtered açai pulp on biomarker of oxidative stress in the liver and hepatic injury markers in rats fed either with control or high-fat diets.

Parameter	Experimental groups			
	C	CA	HF	HFA
TBARS (nmol MDA/mg protein)	0.34 ± 0,017	0.28 ± 0,023	0.48 ± 0,047^*∗*^	0.36 ± 0,040
ALT (IU)	6.86 ± 1.19	11.02 ± 1.43	23.52 ± 1.92^*∗∗∗*^	16.57 ± 1.78^*∗*#^
AST (IU)	18.33 ± 1.19	17.63 ± 1.53	25.03 ± 1.84^*∗*^	20.7 ± 1.05

TBARS, thiobarbituric acid-reactive substances. Values are expressed as means ± SEM (*n* = 8). ^*∗*^Significant at *p* < 0.05 with respect to control group. ^*∗∗∗*^Significant at *p* < 0.001 with respect to control group. ^#^Significant at *p* < 0.05 with respect to High-fat group.

## References

[1] Vernon G., Baranova A., Younossi Z. M. (2011). Systematic review: the epidemiology and natural history of non-alcoholic fatty liver disease and non-alcoholic steatohepatitis in adults. *Alimentary Pharmacology & Therapeutics*.

[2] Wainwright P., Byrne C. (2016). Bidirectional relationships and disconnects between NAFLD and features of the metabolic syndrome. *International Journal of Molecular Sciences*.

[3] Browning J. D., Horton J. D. (2004). Molecular mediators of hepatic steatosis and liver injury. *The Journal of Clinical Investigation*.

[4] Day C. P., James O. F. W. (1998). Steatohepatitis: a tale of two ‘hits’?. *Gastroenterology*.

[5] Rolo A. P., Teodoro J. S., Palmeira C. M. (2012). Role of oxidative stress in the pathogenesis of nonalcoholic steatohepatitis. *Free Radical Biology and Medicine*.

[6] Camps J., Marsillach J., Joven J. (2009). The paraoxonases: role in human diseases and methodological difficulties in measurement. *Critical Reviews in Clinical Laboratory Sciences*.

[7] Précourt L.-P., Amre D., Denis M.-C. (2011). The three-gene paraoxonase family: physiologic roles, actions and regulation. *Atherosclerosis*.

[8] La Du B. N. (1996). Structural and functional diversity of paraoxonases. *Nature Medicine*.

[9] Sözmen E. Y., Sözmen B., Girgin F. K. (2001). Antioxidant enzymes and paraoxonase show a co-activity in preserving low-density lipoprotein from oxidation. *Clinical and Experimental Medicine*.

[10] Draganov D. I., Stetson P. L., Watson C. E., Billecke S. S., La Du B. N. (2000). Rabbit serum paraoxonase 3 (PON3) is a high density lipoprotein-associated lactonase and protects low density lipoprotein against oxidation. *The Journal of Biological Chemistry*.

[11] Mackness M. I., Arrol S., Durrington P. N. (1991). Paraoxonase prevents accumulation of lipoperoxides in low-density lipoprotein. *FEBS Letters*.

[12] Mackness M. I., Arrol S., Abbott C., Durrington P. N. (1993). Protection of low-density lipoprotein against oxidative modification by high-density lipoprotein associated paraoxonase. *Atherosclerosis*.

[13] Ferré N., Camps J., Prats E. (2002). Serum paraoxonase activity: a new additional test for the improved evaluation of chronic liver damage. *Clinical Chemistry*.

[14] Reddy S. T., Wadleigh D. J., Grijalva V. (2001). Human paraoxonase-3 is an HDL-associated enzyme with biological activity similar to paraoxonase-1 protein but is not regulated by oxidized lipids. *Arteriosclerosis, Thrombosis, and Vascular Biology*.

[15] Ng C. J., Wadleigh D. J., Gangopadhyay A. (2001). Paraoxonase-2 is a ubiquitously expressed protein with antioxidant properties and is capable of preventing cell-mediated oxidative modification of low density lipoprotein. *The Journal of Biological Chemistry*.

[16] Shih D. M., Gu L., Xia Y.-R. (1998). Mice lacking serum paraoxonase are susceptible to organophosphate toxicity and atherosclerosis. *Nature*.

[17] Boemi M., Leviev I., Sirolla C., Pieri C., Marra M., James R. W. (2001). Serum paraoxonase is reduced in type 1 diabetic patients compared to non-diabetic, first degree relatives; influence on the ability of HDL to protect LDL from oxidation. *Atherosclerosis*.

[18] Gugliucci A., Kotani K., Kimura S. (2012). Paraoxonase 1 in chronic kidney failure. *Journal of Lipids*.

[19] de Souza M. O., Silva M., de Paula Oliveira R., Pedrosa M. L. (2010). Diet supplementation with acai (*Euterpe oleracea* Mart.) pulp improves biomarkers of oxidative stress and the serum lipid profile in rats. *Nutrition*.

[20] Erlich P. M., Lunetta K. L., Cupples L. A. (2012). Serum paraoxonase activity is associated with variants in the PON gene cluster and risk of Alzheimer disease. *Neurobiology of Aging*.

[21] Ferré N., Marsillach J., Camps J. (2006). Paraoxonase-1 is associated with oxidative stress, fibrosis and FAS expression in chronic liver diseases. *Journal of Hepatology*.

[22] Abdel-Wahhab K. G., Fawzi H., Mannaa F. A. (2016). Paraoxonase-1 (PON1) inhibition by tienilic acid produces hepatic injury: antioxidant protection by fennel extract and whey protein concentrate. *Pathophysiology*.

[23] Kedage V., Muttigi M. S., Shetty M. S. (2010). Serum paraoxonase 1 activity status in patients with liver disorders. *Saudi Journal of Gastroenterology*.

[24] Kilic S. S., Aydin S., Kilic N., Erman F., Aydin S., Celik İ. (2005). Serum arylesterase and paraoxonase activity in patients with chronic hepatitis. *World Journal of Gastroenterology*.

[25] García-Heredia A., Kensicki E., Mohney R. P. (2013). Paraoxonase-1 deficiency is associated with severe liver steatosis in mice fed a high-fat high-cholesterol diet: a metabolomic approach. *Journal of Proteome Research*.

[26] Gong M., Garige M., Varatharajalu R. (2009). Quercetin up-regulates paraoxonase 1 gene expression with concomitant protection against LDL oxidation. *Biochemical and Biophysical Research Communications*.

[27] Varatharajalu R., Garige M., Leckey L. C., Reyes-Gordillo K., Shah R., Lakshman M. R. (2016). Protective role of dietary curcumin in the prevention of the oxidative stress induced by chronic alcohol with respect to hepatic injury and antiatherogenic markers. *Oxidative Medicine and Cellular Longevity*.

[28] Ding L., Li J., Song B. (2016). Curcumin rescues high fat diet-induced obesity and insulin sensitivity in mice through regulating SREBP pathway. *Toxicology and Applied Pharmacology*.

[29] de Oliveira P. R. B., da Costa C. A., de Bem G. F. (2015). *Euterpe oleracea* mart.-derived polyphenols protect mice from diet-induced obesity and fatty liver by regulating hepatic lipogenesis and cholesterol excretion. *PLoS ONE*.

[30] Horton J. D., Goldstein J. L., Brown M. S. (2002). SREBPs: activators of the complete program of cholesterol and fatty acid synthesis in the liver. *The Journal of Clinical Investigation*.

[31] Garige M., Gong M., Varatharajalu R., Lakshman M. R. (2010). Quercetin up-regulates paraoxonase 1 gene expression via sterol regulatory element binding protein 2 that translocates from the endoplasmic reticulum to the nucleus where it specifically interacts with sterol responsive element-like sequence in paraoxonase 1 promoter in HuH7 liver cells. *Metabolism*.

[32] Gouédard C., Barouki R., Morel Y. (2004). Dietary polyphenols increase paraoxonase 1 gene expression by an aryl hydrocarbon receptor-dependent mechanism. *Molecular and Cellular Biology*.

[33] Litvinov D., Mahini H., Garelnabi M. (2012). Antioxidant and anti-inflammatory role of paraoxonase 1: implication in arteriosclerosis diseases. *North American Journal of Medical Sciences*.

[34] Schauss A. G., Wu X., Prior R. L. (2006). Antioxidant capacity and other bioactivities of the freeze-dried Amazonian palm berry, Euterpe oleraceae Mart. (Acai). *Journal of Agricultural and Food Chemistry*.

[35] Marcason W. (2009). What is the açaí berry and are there health benefits?. *Journal of the American Dietetic Association*.

[36] Guerra J. F. C., Magalhães C. L. B., Costa D. C., Silva M. E., Pedrosa M. L. (2011). Dietary açai modulates ROS production by neutrophils and gene expression of liver antioxidant enzymes in rats. *Journal of Clinical Biochemistry and Nutrition*.

[37] Bonomo L. F., Silva D. N., Boasquivis P. F. (2014). Açaí (*Euterpe oleracea* Mart.) modulates oxidative stress resistance in *Caenorhabditis elegans* by direct and indirect mechanisms. *PLoS ONE*.

[38] de Souza M. O., Souza e Silva L., Brito Magalhães C. L. (2012). The hypocholesterolemic activity of açaí (*Euterpe oleracea* Mart.) is mediated by the enhanced expression of the ATP-binding cassette, subfamily G transporters 5 and 8 and low-density lipoprotein receptor genes in the rat. *Nutrition Research*.

[39] Xie C., Kang J., Burris R. (2011). Açaí juice attenuates atherosclerosis in ApoE deficient mice through antioxidant and anti-inflammatory activities. *Atherosclerosis*.

[40] Guerra J. F. C., Maciel P. S., Abreu I. C. M. E. (2015). Dietary açai attenuates hepatic steatosis via adiponectin-mediated effects on lipid metabolism in high-fat diet mice. *Journal of Functional Foods*.

[41] Morán-Ramos S., Avila-Nava A., Tovar A. R., Pedraza-Chaverri J., López-Romero P., Torres N. (2012). Opuntia ficus indica (Nopal) attenuates hepatic steatosis and oxidative stress in obese Zucker (fa/fa) rats. *The Journal of Nutrition*.

[42] William H. (1970). *Official Methods of Analysis*.

[43] Soest Pv. (1963). Use of detergents in the analysis of fibrous feeds. *Journal of the Association of Official Analytical Chemists*.

[44] Georgé S., Brat P., Alter P., Amiot M. J. (2005). Rapid determination of polyphenols and vitamin C in plant-derived products. *Journal of Agricultural and Food Chemistry*.

[45] Giusti M., Wrolstad R. E. (2001). Characterization and measurement of anthocyanins by UV-visible spectroscopy. *Current Protocols in Food Analytical Chemistry*.

[46] Brand-Williams W., Cuvelier M. E., Berset C. (1995). Use of a free radical method to evaluate antioxidant activity. *LWT-Food Science and Technology*.

[47] Reeves P. G., Nielsen F. H., Fahey G. C. (1993). AIN-93 purified diets for laboratory rodents: final report of the American Institute of Nutrition ad hoc writing committee on the reformulation of the AIN-76A rodent diet. *Journal of Nutrition*.

[48] Abreu I. C. M. E., Guerra J. F. C., Pereira R. R. (2014). Hypercholesterolemic diet induces hepatic steatosis and alterations in mRNA expression of NADPH oxidase in rat livers. *Arquivos Brasileiros de Endocrinologia e Metabologia*.

[49] Sun X., Seeberger J., Alberico T. (2010). Açai palm fruit (*Euterpe oleracea* Mart.) pulp improves survival of flies on a high fat diet. *Experimental Gerontology*.

[50] Folch J., Lees M., Sloane-Stanley G. H. (1957). A simple method for the isolation and purification of total lipides from animal tissues. *The Journal of Biological Chemistry*.

[51] Buege J. A., Aust S. D. (1978). [30] Microsomal lipid peroxidation. *Methods in Enzymology*.

[52] Lowry O. H., Rosebrough N. J., Farr A. L., Randall R. J. (1951). Protein measurement with the Folin phenol reagent. *The Journal of Biological Chemistry*.

[53] Brunt E. M., Janney C. G., Di Bisceglie A. M., Neuschwander-Tetri B. A., Bacon B. R. (1999). Nonalcoholic steatohepatitis: a proposal for grading and staging the histological lesions. *The American Journal of Gastroenterology*.

[54] Bayrak T., Bayrak A., Demirpençe E., Kılınç K. (2010). Purification and kinetic properties of rabbit liver paraoxonase 1. *Journal of Chromatography B*.

[55] Cox B., Emili A. (2006). Tissue subcellular fractionation and protein extraction for use in mass-spectrometry-based proteomics. *Nature Protocols*.

[56] Bełtowski J., Wójcicka G., Jamroz A. (2001). Differential effect of 3-hydroxy-3-methylglutaryl coenzyme A reductase inhibitors on plasma paraoxonase 1 activity in the rat. *Polish Journal of Pharmacology*.

[57] Schauss A. G., Wu X., Prior R. L. (2006). Phytochemical and nutrient composition of the freeze-dried amazonian palm berry, *Euterpe oleraceae* mart. (acai). *Journal of Agricultural and Food Chemistry*.

[58] Odendaal A. Y., Schauss A. G., Watson R., Reedy V., Zibadi S. (2014). *Potent Antioxidant and Anti-Inflammatory Flavonoids in the Nutrient-Rich Amazonian Palm Fruit, Açaı (Euterpe spp.)*.

[59] Del Pozo-Insfran D., Brenes C. H., Talcott S. T. (2004). Phytochemical composition and pigment stability of Açai (*Euterpe oleracea* Mart.). *Journal of Agricultural and Food Chemistry*.

[60] Jensen G. S., Wu X., Patterson K. M. (2008). In vitro and in vivo antioxidant and anti-inflammatory capacities of an antioxidant-rich fruit and berry juice blend. Results of a pilot and randomized, double-blinded, placebo-controlled, crossover study. *Journal of Agricultural and Food Chemistry*.

[61] Honzel D., Carter S. G., Redman K. A., Schauss A. G., Endres J. R., Jensen G. S. (2008). Comparison of chemical and cell-based antioxidant methods for evaluation of foods and natural products: generating multifaceted data by parallel testing using erythrocytes and polymorphonuclear cells. *Journal of Agricultural and Food Chemistry*.

[62] Barbosa P. O., Pala D., Silva C. T. (2016). Açai (*Euterpe oleracea* Mart.) pulp dietary intake improves cellular antioxidant enzymes and biomarkers of serum in healthy women. *Nutrition*.

[63] Wong J. M. W., De Souza R., Kendall C. W. C., Emam A., Jenkins D. J. A. (2006). Colonic health: fermentation and short chain fatty acids. *Journal of Clinical Gastroenterology*.

[64] Samy W., Hassanian M. A. (2011). Paraoxonase-1 activity, malondialdehyde and glutathione peroxidase in non-alcoholic fatty liver disease and the effect of atorvastatin. *Arab Journal of Gastroenterology*.

[65] Ustundag B., Bahcecioglu I. H., Sahin K. (2007). Protective effect of soy isoflavones and activity levels of plasma paraoxonase and arylesterase in the experimental nonalcoholic steatohepatitis model. *Digestive Diseases and Sciences*.

[66] Aviram M., Rosenblat M., Billecke S. (1999). Human serum paraoxonase (PON 1) is inactivated by oxidized low density lipoprotein and preserved by antioxidants. *Free Radical Biology and Medicine*.

[67] Kato R., Mori C., Kitazato K. (2009). Transient increase in plasma oxidized LDL during the progression of atherosclerosis in apolipoprotein E knockout mice. *Arteriosclerosis, Thrombosis, and Vascular Biology*.

[68] Subramanian S., Goodspeed L., Wang S. (2011). Dietary cholesterol exacerbates hepatic steatosis and inflammation in obese LDL receptor-deficient mice. *Journal of Lipid Research*.

[69] Bieghs V., Verheyen F., van Gorp P. J. (2012). Internalization of modified lipids by CD36 and SR-A leads to hepatic inflammation and lysosomal cholesterol storage in kupffer cells. *PLoS ONE*.

[70] Walenbergh S. M. A., Koek G. H., Bieghs V., Shiri-Sverdlov R. (2013). Non-alcoholic steatohepatitis: the role of oxidized low-density lipoproteins. *Journal of Hepatology*.

[71] Wouters K., van Gorp P. J., Bieghs V. (2008). Dietary cholesterol, rather than liver steatosis, leads to hepatic inflammation in hyperlipidemic mouse models of nonalcoholic steatohepatitis. *Hepatology*.

[72] Lodovici M., Guglielmi F., Casalini C., Meoni M., Cheynier V., Dolara P. (2001). Antioxidant and radical scavenging properties *in vitro* of polyphenolic extracts from red wine. *European Journal of Nutrition*.

[73] Jarvik G. P., Tsai N. T., McKinstry L. A. (2002). Vitamin C and E intake is associated with increased paraoxonase activity. *Arteriosclerosis, Thrombosis, and Vascular Biology*.

[74] Rock W., Rosenblat M., Miller-Lotan R., Levy A. P., Elias M., Aviram M. (2008). Consumption of wonderful variety pomegranate juice and extract by diabetic patients increases paraoxonase 1 association with high-density lipoprotein and stimulates its catalytic activities. *Journal of Agricultural and Food Chemistry*.

[75] Zagayko A. L., Kravchenko G. B., Krasilnikova O. A., Ogai Y. O. (2013). Grape polyphenols increase the activity of HDL enzymes in old and obese rats. *Oxidative Medicine and Cellular Longevity*.

[76] Chandra S., Sah K., Bagewadi A. (2012). Additive and synergistic effect of phytochemicals in prevention of oral cancer. *European Journal of General Dentistry*.

[77] Aviram M., Billecke S., Sorenson R. (1998). Paraoxonase active site required for protection against LDL oxidation involves its free sulfhydryl group and is different from that required for its arylesterase/paraoxonase activities: selective action of human paraoxonase allozymes Q and R. *Arteriosclerosis, Thrombosis, and Vascular Biology*.

[78] Jaouad L., Milochevitch C., Khalil A. (2003). PON1 paraoxonase activity is reduced during HDL oxidation and is an indicator of HDL antioxidant capacity. *Free Radical Research*.

[79] Khateeb J., Gantman A., Kreitenberg A. J., Aviram M., Fuhrman B. (2010). Paraoxonase 1 (PON1) expression in hepatocytes is upregulated by pomegranate polyphenols: a role for PPAR-*γ* pathway. *Atherosclerosis*.

[80] Deakin S., Leviev I., Guernier S., James R. W. (2003). Simvastatin modulates expression of the PON1 gene and increases serum paraoxonase: a role for sterol regulatory element–binding protein-2. *Arteriosclerosis, Thrombosis, and Vascular Biology*.

[81] Bolanos-Garcia V. M., Miguel R. N. (2003). On the structure and function of apolipoproteins: more than a family of lipid-binding proteins. *Progress in Biophysics and Molecular Biology*.

[82] Rader D. J. (2002). High-density lipoproteins and atherosclerosis. *American Journal of Cardiology*.

[83] Moore R. E., Navab M., Millar J. S. (2005). Increased atherosclerosis in mice lacking apolipoprotein A-I attributable to both impaired reverse cholesterol transport and increased inflammation. *Circulation Research*.

[84] Feio C. A., Izar M. C., Ihara S. S. (2012). *Euterpe oleracea* (açai) modifies sterol metabolism and attenuates experimentally-induced atherosclerosis. *Journal of Atherosclerosis and Thrombosis*.

[85] Androutsopoulos V. P., Kanavouras K., Tsatsakis A. M. (2011). Role of paraoxonase 1 (PON1) in organophosphate metabolism: implications in neurodegenerative diseases. *Toxicology and Applied Pharmacology*.

